# The Effect of Different Milling Methods on the Physicochemical and In Vitro Digestibility of Rice Flour

**DOI:** 10.3390/foods12163099

**Published:** 2023-08-18

**Authors:** Yaning Tian, Lan Ding, Yonghui Liu, Li Shi, Tong Wang, Xueqing Wang, Bin Dang, Linglei Li, Guoyuan Gou, Guiyun Wu, Fengzhong Wang, Lili Wang

**Affiliations:** 1 Institute of Food Science and Technology, Chinese Academy of Agricultural Sciences, Beijing 100193, China; jntianyaning@126.com (Y.T.);; 2 Tibetan Plateau Key Laboratory of Agric-Product Processing, Qinghai Academy of Agricultural and Forestry Sciences, Xining 810016, China; 3 Key Laboratory of Agro-Products Quality and Safety Control in Storage and Transport Process, Ministry of Agriculture and Rural Affairs, Beijing 100193, China

**Keywords:** rice flour, milling methods, particle size, physicochemical properties, starch digestibility

## Abstract

Preparation methods have been found to affect the physical and chemical properties of rice. This study prepared Guichao rice flour with wet, dry, semi-dry, and jet milling techniques. Differences in the particle size distribution of rice flour were investigated in order to assess their impact on pasting, thermal, gel, starch digestibility, and crystalline structure using an X-ray diffractometer (XRD) and a Rapid Visco Analyzer (RVA) across in vitro digestibility experiments. The results showed that semi-dry-milled rice flour (SRF) and wet-milled rice flour (WRF) were similar in damaged starch content, crystalline structure, and gelatinization temperature. However, compared with dry-milled rice flour (DRF) and jet-milled rice flour (JRF), SRF had less damaged starch, a higher absorption enthalpy value, and a higher gelatinization temperature. For starch digestibility, the extended glycemic index (eGI) values of WRF (85.30) and SRF (89.97) were significantly lower than those of DRF (94.47) and JRF (99.27). In general, the physicochemical properties and starch digestibility of WRF and SRF were better than those of DRF and JRF. SRF retained the advantages of WRF while avoiding the high energy consumption, high water consumption, and microbial contamination disadvantages of WRF and was able to produce better rice flour-associated products.

## 1. Introduction

Rice is a basic food on which human beings depend for survival and development. Rice has a light taste and a high yield and is easy to store, making it popular with merchants and consumers [1]. About 60% of global people consume rice as their primary food. Rice contains a high level of carbohydrates and is rich in vitamins and minerals [2]. Since rice is gluten-free, consuming it is considered one of the most effective ways to treat celiac disease (CD) [3,4]. In addition to directly cooking and eating rice, it may be processed into puffed rice and rice flour [5]. Rice flour is extensively processed for various foods, such as bread, noodles, biscuits, and other gluten-free products. Rice flour is made from rice that has been milled. Amylose content in indica rice is higher than that in japonica rice, and amylose molecules age easily and can quickly form into gel, making indica rice more suitable for making rice flour and rice pasta [6].

During the preparation of rice flour, the milling of raw rice is the most important step, and milling methods mainly include wet, dry, semi-dry, and jet milling. Soaked samples can be wet-milled with water and then dried into rice flour [7]. Soaking significantly affects the qualities of wet-milled rice flour (WRF). For example, soaking reduces the hardness of rice and helps achieve a finer particle size and delicate texture in WRF, thus increasing the quality and yield of rice flour products [5,8]. Another commonly used method is the dry milling method. Dry-milled rice flour (DRF), which is low in moisture content and has a long storage life, can be produced through direct milling and sieving [8]. However, dry milling affects the distribution of different particle sizes in rice flour and causes damage to starch particles, which can lead to changes in the processing properties of rice flour products [9]. To avoid high levels of damaged starch in dry milling and high energy consumption, water consumption, nutrient loss, and microbial contamination during wet milling, semi-dry milling has been used in grain milling [10]. Soaked rice and milling are semi-dry processing methods, and semi-dry-milled rice flour (SRF) has similar properties to WRF [11]. Jet milling technology is a new food processing technology that is extensively applied in the food, cosmetics, medicine, metallurgy, and aerospace fields [4]. Compared with traditional mechanical processing methods, jet-milled rice flour (JRF) has higher degree of comminution, certain interfacial activity, an increased specific surface area, reduced interfacial tension, as well as an improved color, flavor, and texture [12]. Different milling methods can cause differences in particle size and damaged starch content, which can affect the processing characteristics of rice-based foods [10]. Wang et al. (2020) concluded that damaged starch can influence the gelatinization, pasting, and in vitro digestibility of starch-based foods [13].

Because of the high starch content in rice flour, starch digestibility has a significant influence on the biochemical indicators of human blood glucose [14]. This may lead to diet-related health complications like type 2 diabetes [15]. Therefore, the starch digestibility of rice flour is of increasing interest. The lower degree of hydrolysis can effectively reduce the rise in blood glucose after meals [16]. Hosson et al. (2015) compared the digestibility of hammer-milled flour, jet-milled flour, and submicron-scale WRF and suggested that submicron-scale WRF was more easily digested [17]. Lee et al. (2019) compared hammer milling and air jet milling, using germinated brown rice as the sample. Coarse fraction jet-milled flour exhibited higher starch hydrolysis levels [18]. It can be concluded that milling methods influence the digestibility of rice flour starch by varying the particle size, damaged starch content, and crystalline structure [16]. Therefore, it is of interest to research the impact of different four preparations and particle sizes for the in vitro digestion of rice flour.

Rice flour is the main material used for rice-based foods, and the physicochemical properties and digestibility of raw materials determine food quality, so it is essential to study the preparation of raw materials. This study aims to investigate the impact of four preparation methods on the particle size distribution and its effect on the damaged starch content, pasting, thermal and gel properties, and starch digestibility of flour. This study expects to obtain low GI rice flour with better physicochemical properties that can enhance the quality of rice flour-related products.

## 2. Materials and Methods

### 2.1. Materials

Guichao rice (early indica rice) was bought from Jiangxi Huadachang Food Industry Co., Ltd. (Ganzhou, China) in 2019. The water content was 8.08 g/100 g, the protein content was 8.36 g/100 g, the lipid content was 0.73 g/100 g, the ash content was 0.77 g/100 g, the total starch content was 84.04 g/100 g, and the amylose content was 21.61 g/100 g. The procured rice was kept at −20 °C before experimental use. The porcine pancreas α-amylase, pepsin, pancreatin, and amyloglucosidase (AMG) were obtained from Sigma-Aldrich Trading Co., Ltd. (Shanghai, China). The D-Glucose Assay Kit (GOPOD format), Total Starch Assay Kit, Starch Damage Assay Kit, and Amylose Assay Kit were procured from Megazyme International Ireland Ltd. (Wicklow, Ireland). The chemicals employed in this research were of analytical quality.

### 2.2. Preparation of Rice Flour

WRF: In 4 kg of deionized water, 2 kg of sample was soaked at 25 °C for 6–8 h. The soaked rice was mixed with 1 kg of distilled water and coarsely milled in a colloid mill (JMS-30A, Langfang Langtong Machinery Co., Ltd., Langfang, China). The coarse pulverized pulp was then milled for 3 cycles with a gap of 0.10–0.15 mm [19]. Thereafter, the rice pulp was freeze-dried and passed through standard sieves of 80 mesh and stored in a hermetic bag at 4 °C until use.

SRF: A total of 2 kg of the sample was mixed with the required amount of water and soaked at 25 °C. The sample was sealed and shaken, then left to be tempered at 25 °C. The rice was tempered for 8 h, during which time the rice was shaken every hour to adjust the moisture content to 27%. The rice was then crushed in a cyclone mill (CT410, FOSS Scino [Suzhou] Co., Ltd., Suzhou, China) and passed through standard sieves of 80 mesh [19]. The milled rice flour was roasted at 40 °C until water content was below 12%, then kept in a hermetic bags at 4 °C until needed.

DRF: A total of 5 kg of rice was milled in a cyclone mill (CT410, FOSS Scino (Suzhou) Co., Ltd., Suzhou, China) and was passed through standard sieves of 80 mesh [20]. The DRF was stored in a hermetic bag at 4 °C for use.

JRF: A total of 5 kg of rice was milled using an airflow ultrafine mill (Weifang Jinghua Powder Engineering Equipment Co., Ltd., Weifang, China) and was passed through 200 mesh sieve and were stored in a hermetic bag at 4 °C for use [18].

### 2.3. Physicochemical Compositions

The contents of moisture, crude protein (GB 5009.5-2016), crude fat (GB 5009.6-2016), and ash (GB 5009.4-2016) were determined according to the National Standards of China. The contents of total starch, amylose, and damaged starch were determined using a Total Starch Assay Kit, Amylose/Amylopectin Kit, and Starch Damage Assay Kit (Megazyme International Ltd., Wicklow, Ireland), respectively.

### 2.4. Crystalline Structure of Rice Flour

An X-ray diffractometer (D2 PHASER, Bruker AXS, Karlsruhe, Germany) was used to determine the crystalline structure of samples. The determination method was carried out according to the method of Wang et al. (2018) [21]. An appropriate amount of rice flour was placed in a desiccator (with an internal relative humidity of 100%) and the moisture was equilibrated for 48 h. The equilibrated rice flour was pressed in the grooves of the glass sheet so that the sample surface was smooth and level with the glass sheet surface. The sample was scanned using a Cu–Kα radiation source of 40 kV and 40 mA. The scanning range was 5–45° (2θ), the step width was 0.0131°, and the speed was 2°/min [16].

### 2.5. Determination of Particle Size Distribution

A Malvern laser particle size analyzer (Mastersizer 3000, Malvern Instruments, Worcestershire, UK) was used to measure the particle size distribution of the dry powder [20]. D10, D50, and D90 were calculated using the software supplied with the device [22].

### 2.6. Pasting Properties of Rice Flour

The pasting properties of the samples were determined using a Rapid Visco Analyzer (RVA4500, Perten Instruments, Macquarie, NSW, Australia). Measurements were based on the method of Gao et al. (2019) [23] with some adaptations. A total of 3.50 g of sample (14 g/100 g water) was weighed out and distilled water was added to the RVA jar. The amount of distilled water to be added was identified using the instrument and corresponded to the moisture content of samples. The determination was made according to the standard RVA procedure: the samples were maintained at 50 °C for 1 min, warmed to 95 °C in 198 s, equilibrated at 95 °C for 2.9 min, reduced to 50 °C in 229 s, and then maintained for 1 min. During this process, Samples were blended with a paddle speed of 960 rpm for 10 s, all other time was 160 rpm [3].

### 2.7. Thermal Properties

A differential scanning calorimeter (DSC-Q200, TA Instruments, New Castle, TL, USA) was used to determine the thermal properties of a sample. The method of determination was modeled on the method of Li et al. (2017) [24] with some adaptations. A total of 3 mg of the sample was added to the crucible, and distilled water 3 times its weight was added. The samples were sealed and left overnight at 4 °C as blank controls. Determination was carried out according to the standard DCS-Q200 procedure: the thermal properties curve was measured by heating from 35 °C to 110 °C at 10 °C/min. The curves were analyzed to obtain the onset temperature (To), peak temperature (Tp), conclusion temperature (Tc), and enthalpy change (*ΔH*).

### 2.8. Gel Properties of Rice Flour

When added to an amount of water, 10 g of rice flour formed a suspension of 15% mass fraction. The determination method was based on the method of Li et al. (2017) [24]. The mixture was stirred at 35 °C for 1 min and placed in a water bath at 95 °C for 30 min. The gel was left to cool at 4 °C overnight. The gel properties of the sample were measured using a texture analyzer (TA-XT 2i/5, Stable Micro System Ltd., Godalming, UK) with a P/36R probe in texture profile analysis (TPA) mode [4]. The measurement speed of pre-test and test was 0.5 mm/s. The compression distance was 10.0 mm, the triggering force was 5.0 g, and the compression interval was 3 s [4].

### 2.9. Determination of Starch Digestibility

The methods of Syahariza et al. (2013) [25] and Zou et al. (2015) [26] with some modifications were used to determine starch digestibility. This method simulated in vitro the digestion of starch in the mouth, stomach, and small intestine. A sample equivalent to 50 mg (on a dry basis) of rice starch was added to 2 mL of distilled water and 3 glass beads. The samples were dispersed and mixed with a vortex oscillator, then boiled in a water bath for 20 min and cooled. The samples were placed in a 37 °C thermal shaker incubator (MTH-100, Hangzhou Miu Instrument Co., Ltd., Hangzhou, China) with a 5000 rpm shake rate. The 1 mL of α-amylase (Sigma A3176) from the porcine pancreas was dropped into the suspension, and warmed at 37 °C for 2 min. A total of 5 mL of pepsin (Sigma P6887, pH 2.0) was added and warmed at 37 °C for 30 min. The digest was neutralized by 5 mL of 0.02 M NaOH, and the pH value was adjusted via 20 mL of sodium acetate buffer. A total of 5 mg of pancreatin (Sigma P1750) and 43µL of amyloglucosidase (Sigma A7095) were added to the digesta [16]. The measurement of the glucose concentration was carried out at specific times, namely 10, 20, 30, 40, 50, 60, 90, 120, 180, and 140 min, and then dispersed into 1 mL of 95% (*v*/*v*) ethanol to terminate the reaction. In addition, 0.1 mL of glucose (1 mg/mL) was taken as the standard sample, while 0.1 mL of 95% (*v*/*v*) ethanol was used as the blank control. Each analysis was repeated three times. Starch digestibility was obtained using the following equation:

(1) $$Dt=A\times \frac{0.1\times 1}{As}\times \frac{V\times N}{B}\times 10\times \frac{162}{180}$$

where *A* is the absorbance per time point, and *As* (*A* sample) is the absorbance of the standard. *V* is the volume of the digestive solution, *N* is the dilution multiple, and *B* is the basic dry mass of the sample. The $\frac{162}{180}$ expresses the weight coefficient of the starch (monomeric unit anhydroglucose) converted to glucose [26].

The starch hydrolysis curves were plotted based on the glucose content of samples sampled at specific time points. The starch hydrolysis rate curves often followed first-order kinetics, as indicated by Equation (2):

(2) $$C_{t}=C_{\infty }\left(1-e^{-kt}\right)$$

where *C_t_* expresses the glucose concentration at digestive time *t*, *C_∞_* is the glucose concentration at the final timepoint (240 min), and *k* is the digestibility coefficient [27].

The area under curve (*AUC*) was obtained from the *k* value, as shown in Equation (3):

(3) $$AUC=C_{\infty }\left(t-t_{0}\right)-\frac{C_{\infty }}{k}\left[1-{exp}^{-k\left(t-t_{0}\right)}\right]$$

The hydrolysis index (*HI*) is the ratio of the sample *AUC* to the standard sample *AUC* [16], as shown in Equation (4):

(4) $$HI=\frac{AUC_{sample}}{AUC_{standard\;sample}}\times 100\%$$

The calculation of *eGI*, according to the method of Granfeldt et al. (1992) [28], is shown in Equation (5):

(5) eGI=0.862×HI+8.198

### 2.10. Statistic Analyse

Each sample was duplicated three times and the average value was obtained. All data were processed using Origin (version 2018, Stat-Ease Inc., Minneapolis, MN, USA) software and SPSS (version 16.0, SPSS Inc., Chicago, IL, USA). 

## 3. Results and Discussion

### 3.1. Particle Size Distribution of Rice Flour

The particle size distribution of grain flour affected the physicochemical properties and processing qualities of sample, which further affected the edibility and sensory quality of products [29]. The particle size distribution of sample under different methods is shown in Table 1. As shown, the milling method had a great influence on the particle size. JRF had a narrow particle size distribution (4.07–29.15 μm) with an average particle size of 14.65 μm, which was significantly smaller than that obtained by other milling methods. This was due to the full impact of the material driven by the high speed airflow during jet milling, making the rice flour finer and more uniformly distributed [4]. The average particle size of WRF was 23.43 μm, of SRF it was 39.10 μm, and of DRF it was 59.45 μm. Among several milling methods, DRF had the widest particle size distribution (13.30 to 154.50 μm) with more large particles. The average particle size of WRF and SRF were close to each other, but SRF had a slightly higher average particle size than WRF.

**Figure 1:**
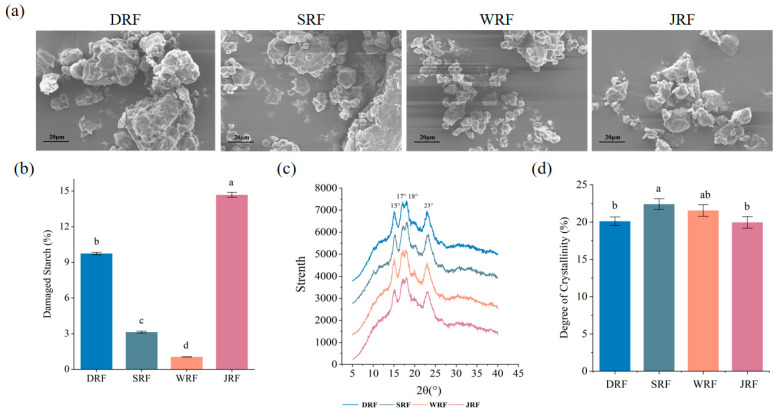
Scanning electron microscope images of samples (**a**), damaged starch content (**b**), X-ray diffraction (**c**), and relative crystallinity of rice flour using four milling methods (**d**). Values with different letters in the figure are significantly different (*p* < 0.05).

The physicochemical properties and digestibility of sample were largely influenced by the particle size of rice flour [6]. When comparing the average particle size of the four types of rice flour, it can be seen that WRF has fine particles, SRF has medium particles, DRF has coarse particles, and JRF has ultrafine particles. From Figure 1a, it can be seen that four types of rice flour were irregularly polyhedral-shaped. This is in agreement with the observations of Farooq et al. [6]. SRF and WRF had smoother surfaces. DRF had larger particles and rougher surfaces. The ultrafine JRF particles had fewer angles but a rougher surface.

### 3.2. Damaged Starch Content and Relative Crystallinity of Rice Flour

During rice milling, starch particles can be damaged and even starch molecular structure can be degraded, thus increasing the amount of damaged starch [30]. The main factor affecting the processing characteristics of rice flour was damaged starch content, followed by particle size distribution [31]. By comparing the effects of four preparation types on the damage degree of sample (see Figure 1a), evidently, damaged starch content varied significantly among four samples. Among them, WRF had a more granular structure and less damaged starch content, followed by SRF. This indicated that pre-moistening treatment in the semi-dry milling process effectively reduced the damaged starch content. One reason for this could be that the rice absorbed enough moisture to be easily milled, thus reducing damaged starch content. On the other hand, it could be related to mechanical and thermal energy [32]. The liquid absorbed the heat generated during the milling process and reduced the thermal damage to the starch [31]. The dry-milling process generated higher mechanical and thermal energy, resulting in more damaged starch content [11]. Although the heat generated by material friction in the jet-milling process was reduced, the degree of damaged starch content was the highest. This was due to the mechanical force generated by the high intensity impact that destroyed the starch structure [18]; thus, the damaged starch content reached 14.56%. Overall, the mechanical forces and heat in the milling step significantly influenced the degree of damage to the rice flour.

X-ray diffraction was used as an analytical method to study the crystalline structure of starch. From Figure 1b, it can be seen that four rice flours had obvious peaks at 15°, 17°, 18°, and 23°. Among them, the connected double peaks near 17° and 18° were typical A-type starch crystals. It can be inferred that the starch crystalline type was A-type [33,34], and the rice starch crystals remained the same, despite the different methods of milling. This indicated that the crystalline structure of rice starch was not changed by mechanical forces and different milling methods. The same result was obtained by Marti et al. (2010) [33]. In addition, it can be seen that the peak shapes of the SRF and WRF are particularly similar. The starch structure of the WRF and SRF was more similar to that of DRF and JRF.

The crystallinity degree of rice flour obtained under various milling conditions is presented in Figure 1c. It was found that the crystallinity degree of rice flour produced under different milling techniques was similar (*p* > 0.05). The largest difference in crystallinity degree was between SRF and JRF, with a difference of 2.45%, while the smallest difference in crystallinity degree was between DRF and JRF, with a difference of 0.15%. This indicated that the various flour processing techniques had little influence on the crystallinity of rice starch.

### 3.3. Pasting Properties

Rice flour cannot form a gluten network structure, and the main structure of rice flour products consists mainly of starch gels. Therefore, it is crucial to investigate the effect of various production processes on the pasting properties of sample, which to some extent reflected the quality of products [35]. The results are shown in Table 2, the RVA analysis showed the change in the viscosity of rice flour during heating and cooling. The pasting temperature was 80.67 °C for WRF and only 72.68 °C for JRF, which was consistent with the findings of Wu et al. (2019) [4]. This can be associated with damaged starch content. When damaged starch granules were combined with water, they swelled rapidly and gelled easily at lower temperatures [22]. Peak viscosity was an important index for measuring the water-holding capacity of starch [3]. Increased peak viscosity reflected that the starch granular structure no longer supported swelling and, therefore, that the starch granular structure was damaged and had begun to deform and break. It can be seen that the peak viscosity of samples was negatively related to damaged starch. Compared to SRF and WRF, DRF and JRF had high damaged starch content and low peak viscosity (*p* < 0.05). The starch molecules were heated and gelatinized to form a viscous gel [36]. On cooling to 50 °C, the final viscosity of rice flours began to increase after cooling, indicating the formation of a rigid gel structure upon cooling. Residual starch granules interacted with leached molecules and formed a network structure, contributing to the increase in viscosity during cooling [37]. JRF had the lowest breakdown viscosity, which meant that the gel it formed was more able to withstand heating and shearing [3]. Interestingly, the peak and final viscosities of WRF and SRF were significantly higher than those of DRF and JRF (*p* < 0.05), suggesting that the water-holding capacity and network structure of these two flours were improved [8,10]. The setback value can be used to measure the degree of starch aging and regeneration. WRF and DRF had the highest regeneration values, followed by SRF; however, all were significantly higher than JRF. This suggested that they have a stronger gelling ability than JRF [4].

### 3.4. Thermal Properties

When a phase change of the material occurs, it is always accompanied by energy absorption or release. In the DSC analysis, the crystalline structure of starch absorbs heat during the melting process, which appears as a heat absorption peak. The larger the peak area, the more heat is required to melt the crystal and the higher the melting enthalpy change. As shown in Table 2, the milling method had a great influence on the thermal properties. The DSC results indicated that the gelatinization of rice starch occurred at a temperature range of 64.68 to 69.54 °C. To, Tp, and Tc in DRF were obviously larger than in SRF and WRF, which suggests that rice flour with particles of large size can not easily be pasted [10]. When the temperature reached the onset temperature of rice flour pasting, small particles combined with water to start gelatinization, while large particles were not able to react as quickly, so the gelatinization temperature was higher than in flour with small particles [5]. The damaged starch and particle size in SRF were closer to those in WRF, so there was no significant difference in To and Tc between SRF and WRF. Interestingly, the JRF had the smallest median particle size, but the highest To, Tp, and Tc. The analysis of the data in Table 2 shows that To, Tp, and Tc were positively correlated with damaged starch content. Compared to the other three milling methods, JRF had the highest damaged starch content with significantly higher To, Tp, and Tc values (*p* < 0.05). However, one study found that samples with smaller particle sizes and more broken starch had lower Tp and Tc values [38]. This could be due to the effect of non-starch ingredients (e.g., lipids and protein) on the thermal properties of the flour, such as the denaturation of protein and the interaction of starch with fat or protein [38,39]. In addition, in samples with more damaged starch, the aggregation of starch granules affected the starch–water system formation and gelatinization temperature [40]. *ΔH* reflects the energy of the transition from a crystalline to a non-crystalline structure [6]. There was a significant difference in the *ΔH* of rice flour between the various milling techniques (*p* < 0.05). The different milling methods resulted in large differences in damaged starch content. WRF had a maximum *ΔH* because WRF had less damaged starch and relatively intact granules, thus requiring more energy to break the granule structure and absorbing more heat in the process [4]. The *ΔH* was negatively correlated with damaged starch content, so JRF had the lowest *ΔH* value of the four samples. In general, the thermal properties of the four samples were associated with changes in particle size and damaged starch content due to preparation methods [38].

### 3.5. Gel Properties

The texture of products was influenced by the gel properties of the samples. TPA results are shown in Table 3. DRF and WRF were significantly different in all indicators (*p* < 0.05). Compared to WRF, SRF, DRF, and JRF showed increased resilience, springiness, and chewiness. In terms of hardness, DRF had the highest hardness of 6.96 N, followed by SRF and, finally, WRF and JRF. The gel hardness of WRF was close to that of JRF (*p* > 0.05). In terms of the adhesiveness index, the viscosity of the other three flours was higher than that of WRF. DRF had the highest hardness, and this was attributed to damaged starch content. Rice flour was easy to setback when broken starch was high. The setback was due to increased adhesiveness caused by the rearrangement of amylose during cooling [41]. High DRF setback values indicated a high degree of recrystallisation and an increase in gel hardness [42,43]. Although JRF had high damaged starch content, its setback values, adhesiveness, and hardness were different from those of DRF. Qiu et al. (2019) concluded that the milling method could change the amylose content in samples [44]. The authors of some studies suggested that this could be due to the non-starch components of rice flour, and starch and lipids formed starch–lipid complexes [40]. Some studies have shown that lipids inhibited starch granule swelling and reduced granule setback value and hardness when gelling [40,45]. In addition, samples with high damaged starch content had poorer gel properties after pasting, and damaged starch content in the sample should be kept at a low level to ensure the gel properties of rice products [46].

### 3.6. Starch Digestibility

**Figure 2:**
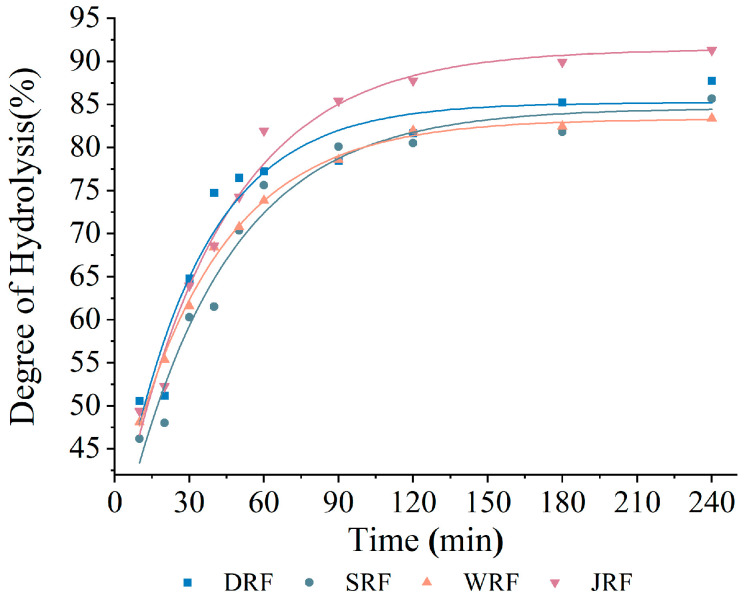
Hydrolysis curves of samples under four milling methods.

Many kinds of cereals were crushed before production and processing. The degree of milling and the size of the flour not only affected the processing properties, such as water absorption and solubility, but also affected the digestibility of the rice flour products. Figure 2 shows that the milling method had a significant influence on the starch hydrolysis of rice flour. JRF exhibited the highest hydrolysis degree (90.59%), followed by DRF (87.20%), SRF (85.10%), and WRF (83.78%). This phenomenon could be related to particle size and damaged starch content. In JRF, the particle size was the smallest and damaged starch content was the highest. This increased the surface area of the particles and made it easier to contact and react with digestive enzymes, thus increasing the amount of hydrolysis products [16,18]. Table 4 shows the in vitro starch hydrolysis indexes of rice flour under different milling techniques. The analysis of data revealed that JRF had the highest K, C∞, AUC, and eGI values, followed by DRF, SRF, and WRF. Starch hydrolysis indexed with the increase in damaged starch content. [46]. Different milling methods affected the surface area, gaps, and cracks of the rice flour particles, thus affecting the digestibility of rice flour [47]. More damaged starch content and larger gaps allowed for more contact area between digestive enzymes and particles. The gaps were more convenient for digestive enzymes to enter into cells for the reaction, causing the rice flour particles to be more thoroughly digested. As a result, the value of various digestive indexes was higher. JRF had smaller particles and the highest damaged starch content, resulting in higher water diffusion and a larger surface area for contact with digestive enzymes [48]. Therefore, the four indexes of starch hydrolysis were significantly higher than those of the other rice flour samples. In addition, different milling methods may result in differences in amylose, amylopectin content, and differences in non-starch components, such as lipids. All these could affect the digestibility of samples [6,16,18,49]. It is noteworthy that the eGI of SRF and WRF was significantly lower than that of DRF and JRF (*p* < 0.05). This may be due to the semi-dry milling method and the wet milling method retaining more intact cell walls in the rice flour. The cell walls reduced the contact area for digestive enzymes and increased the barrier to enzyme entry into the cell [6]. The action area of digestive enzymes on rice flour particles was reduced and, thus, the eGI of the samples was lower. Low eGI can effectively control blood glucose and provides health benefits. Generally, the eGI of rice flour products is higher than that of other cereals, and reducing the eGI of rice flour products is a major challenge in production [49]. The results of in vitro digestion experiments suggest that it is feasible to reduce the eGI of samples using the four milling methods.

## 4. Conclusions

This research compared the effects of dry, semi-dry, wet, and jet milling methods on the particle size, physicochemical properties, and digestibility of rice flour. The results indicate that milling methods had no effect on the crystalline structure of samples. Compared with DRF and JRF, SRF was close to WRF in terms of damaged starch content, crystalline structure, and gelatinization temperature and had a better network structure. DRF had high degree of recrystallisation and the highest gel hardness. In terms of rice flour digestibility, the eGI of SRF was significantly lower than that of DRF and JRF, which was close to that of WRF. In general, WRF and SRF had better physicochemical properties and starch digestibility. Due to the high energy consumption and water consumption of WRF, SRF is more suitable for the preparation of low-eGI processed rice flour products. In future studies, semi-dry milled rice flour will be used to prepare various types of rice flour products with a lower glycemic index, such as rice noodles and rice cakes, and to assess their starch digestibility in vivo and in vitro.

## Figures and Tables

**Table 1:** Particle size distribution of samples using four milling methods.

Sample	DRF	SRF	WRF	JRF
D10 (μm)	13.30 ± 0.00 ^a^	9.66 ± 0.47 ^b^	7.68 ± 0.12 ^c^	4.07 ± 0.06 ^d^
D50 (μm)	59.45 ± 0.21 ^a^	39.10 ± 0.09 ^b^	23.43 ± 0.09 ^c^	14.65 ± 0.07 ^d^
D90 (μm)	154.50 ± 0.71 ^a^	96.15 ± 4.41 ^c^	107.93 ± 2.52 ^b^	29.15 ± 0.35 ^d^

WRF—wet-milled rice flour; SRF—semi-dry-milled rice flour; DRF—dry-milled rice flour; JRF—jet-milled rice flour. Values with different letters in the table are significantly different (*p* < 0.05).

**Table 2:** Pasting and thermal properties of samples under four milling methods.

Sample	DRF	SRF	WRF	JRF
Pasting Properties				
Pasting Temperature (°C)	73.50 ± 0.05 ^c^	77.12 ± 0.94 ^b^	80.67 ± 0.06 ^a^	72.68 ± 1.17 ^c^
Peak Viscosity (cP)	5466 ± 60 ^b^	5667 ± 25 ^a^	5613 ± 137 ^a^	4960 ± 23 ^c^
Though Viscosity (cP)	3443 ± 100 ^c^	3881 ± 102 ^a^	3822 ± 117 ^a^	3581 ± 49 ^b^
Final Viscosity (cP)	6705 ± 47 ^c^	6863 ± 61 ^b^	7100 ± 106 ^a^	6118 ± 7 ^d^
Breakdown (cP)	2023 ± 43 ^a^	1786 ± 88 ^b^	1790 ± 34 ^b^	1379 ± 25 ^c^
Setback (cP)	3262 ± 60 ^a^	2982 ± 48 ^b^	3278 ± 23 ^a^	2536 ± 56 ^c^
Thermal Properties				
To (°C)	61.32 ± 0.09 ^b^	60.75 ± 0.12 ^c^	59.29 ± 0.27 ^c^	64.14 ± 0.06 ^a^
Tp (°C)	67.07 ± 0.17 ^b^	66.02 ± 0.09 ^c^	64.68 ± 0.13 ^d^	69.54 ± 0.00 ^a^
Tc (°C)	71.46 ± 0.51 ^b^	70.14 ± 0.24 ^c^	69.09 ± 0.20 ^c^	74.69 ± 0.08 ^a^
*c* (J/g)	5.75 ± 0.07 ^c^	7.85 ± 0.13 ^b^	8.21 ± 0.06 ^a^	4.12 ± 0.10 ^d^

To: onset temperature; Tp: peak temperature; Tc: conclusion temperature; ΔH: enthalpy change. Values with different letters in the table are significantly different (*p* < 0.05).

**Table 3:** Gel properties of samples under four milling methods.

Sample	DRF	SRF	WRF	JRF
Hardness (N)	6.96 ± 0.42 ^a^	5.37 ± 0.20 ^b^	3.50 ± 0.15 ^c^	3.74 ± 0.07 ^c^
Adhesiveness(N.s)	−17.38 ± 3.39 ^c^	−14.40 ± 2.23 ^bc^	−5.65 ± 0.64 ^a^	−11.87 ± 1.25 ^b^
Resilience (%)	4.21 ± 0.82 ^b^	5.48 ± 0.49 ^a^	2.49 ± 0.68 ^c^	4.08 ± 0.75 ^b^
Cohesiveness	0.54 ± 0.03 ^b^	0.52 ± 0.01 ^b^	0.42 ± 0.03 ^c^	0.58 ± 0.01 ^a^
Springiness (%)	94.96 ± 6.18 ^a^	87.76 ± 6.40 ^b^	84.48 ± 8.38 ^b^	96.03 ± 2.92 ^a^
Gumminess	384.01 ± 41 ^a^	286.04 ± 8 ^b^	150.31 ± 15 ^d^	220.81 ± 6 ^c^
Chewiness	362.38 ± 37 ^a^	250.86 ± 16 ^b^	127.93 ± 26 ^c^	212.14 ± 11 ^b^

Values with different letters in the table are significantly different (*p* < 0.05).

**Table 4:** Digestibility of samples under four milling methods.

Sample	DRF	SRF	WRF	JRF
*K* (×10^−2^)	2.99 ± 0.04 ^b^	2.54 ± 0.15 ^c^	2.08 ± 0.01 ^d^	3.32 ± 0.05 ^a^
*C*∞ (%)	87.20 ± 0.61 ^b^	85.10 ± 0.92 ^c^	83.78 ± 0.38 ^d^	90.59 ± 0.66 ^a^
*AUC*	180.15 ± 1.65 ^b^	170.75 ± 1.28 ^c^	161.00 ± 0.93 ^d^	189.62 ± 1.12 ^a^
*eGI*	94.47 ± 0.79 ^b^	89.97 ± 0.61 ^c^	85.30 ± 0.45 ^d^	99.27 ± 0.59 ^a^

Values with different letters in the table are significantly different (*p* < 0.05).

## Data Availability

Research data are not shared.
